# Pancreatic Cancer Heralded by Worsening Glycemic Control: A Report of Two Cases

**DOI:** 10.1177/2324709617714286

**Published:** 2017-06-08

**Authors:** Dimpi Desai, Devika Rao, Vineeth Sukrithan, Eleanor Weinstein, Akankasha Goyal, Ulrich Schubart

**Affiliations:** 1 Jacobi Medical Center, Bronx, NY, USA; 2 Montefiore Hospital and Medical Center, Bronx, NY, USA; 3 NYU Langone Medical Center, New York, NY, USA

**Keywords:** diabetes mellitus type 2, pancreas, cancer

## Abstract

Pancreatic ductal adenocarcinoma is the third leading cause of cancer-related death in the United States. Since it is usually diagnosed at an advanced stage, its prognosis remains poor. The initial presentation varies according to the tumor location. The most common presenting signs are weight loss, jaundice, and pain. Several epidemiological, clinical, and experimental studies over the past 2 decades have shown that long-standing diabetes is a modest risk factor for pancreatic cancer. However, new-onset diabetes has also been observed to be an early manifestation of pancreatic cancer. We report 2 cases where worsening glycemic control led to the diagnosis of pancreatic cancer.

## Case 1

A 96-year-old woman, with a history of hypertension, diet-controlled diabetes mellitus, hyperlipidemia, chronic kidney disease, and gastroesophageal reflux presented to the emergency room with sudden onset of confusion. As per her daughter, the patient had been unable to recognize her family members in the past day, which was significantly different from her baseline behavior. The daughter also noticed polyuria but no fever, chills, diarrhea, abdominal pain, or dysuria. Vital signs on admission revealed a temperature of 98.5°F, blood pressure of 166/87 mm Hg, heart rate of 91 beats per minute, respiratory rate of 22 breaths per minute, saturating at 99% on room air, with a finger-stick glucose reading >500. The physical exam was unremarkable. A high glucose level was confirmed on serum chemistry (Table 1), with additional findings of normal serum bicarbonate (26.5 mEq/L) and trace ketones in the urine. In this setting of elevated glucose, minimal ketonemia, absence of acidosis, and change in mental status, a diagnosis of hyperglycemic hyperosmolar state (HHS) was made. As per the American Diabetes Association guidelines for the management of HHS, she was treated with intravenous fluids and an insulin drip until normalization of blood glucose was achieved and then transitioned to subcutaneous insulin. An investigation of precipitating factors of HHS did not reveal any obvious cause. During the hospital stay, her symptoms improved and she was discharged home on long-acting basal insulin glargine for glycemic control.

**Table 1.** Laboratory Parameters of Case 1.

Parameter (Normal Range)	Prior to 6 Months of Admission	At the Time of Admission	One Month After Discharge
Hemoglobin A1c (3.9% to 6.9%)	6.7	11	11.8
Glucose (70-105 mg/dL)	115	1037	422
Osmolality (275-295 mOsm/kg)		363	
Total bilirubin (0.1-1.2 mg/dL)	0.4	0.4	20.6
Direct bilirubin (0-0.3 mg/dL)			17.1
Aspartate transaminase (1-40 U/L)	21	29	397
Alanine transaminase (1-40 U/L)	12	19	354
Alkaline phosphatase (30-115 U/L)	118	154	1481

**Figure 1.**
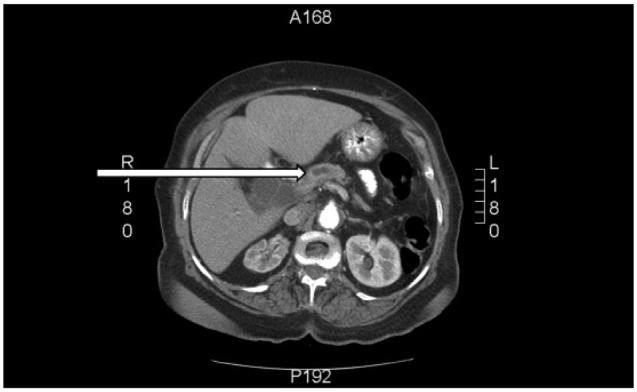
CT scan image of Case 1.

**Figure 2.**
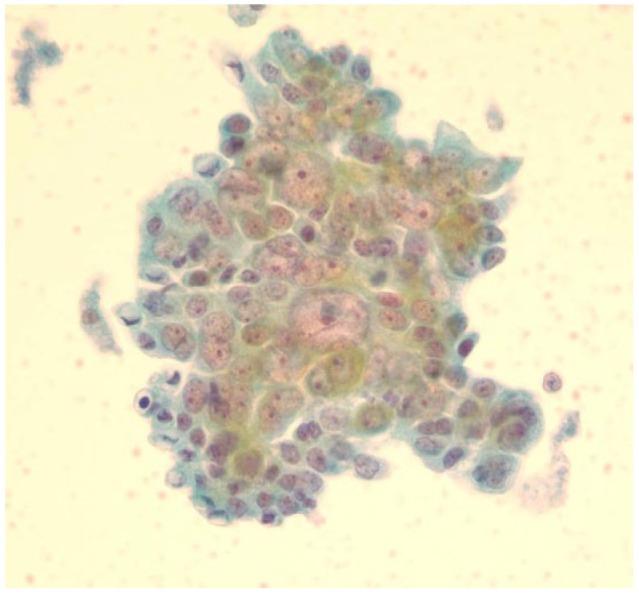
Pathology slide image of Case 1.

The patient was followed in the primary care clinic 2 weeks after discharge, where she was noted to have elevated postprandial blood glucose levels with normal fasting blood glucose levels. Prandial insulin was then added to her regime. Within a month after discharge, the patient reported darkening of her urine, clay colored stools, pruritus, and yellow discoloration of her skin. She also reported fatigue, poor appetite, and unintentional weight loss. Laboratory investigations were significant for obstructive jaundice (Table 1) and negative for infectious hepatitis. An ultrasound of the abdomen revealed distension of the intrahepatic ducts with a “double duct sign,” which prompted further evaluation for distal obstructing lesions. A computed tomography (CT) scan of the abdomen showed dilatation of the biliary and, to a lesser extent, pancreatic tree, with an abrupt cutoff of ducts around the pancreatic head (Figure 1). This portion of the pancreas exhibited ill-defined hypo-attenuation. A biopsy of tissue from this area confirmed the diagnosis of pancreatic ductal adenocarcinoma (PDAC; Figure 2). An endoscopic retrograde cholangio-pancreatography was performed, which revealed a 3.6 × 1.5 cm irregularly shaped pancreatic head/neck mass. The portal veins and hepatic artery were in close relation to the mass with multiple enlarged lymph nodes as well. It was deemed to be an inoperable PDAC and a metal stent was placed at the site of biliary obstruction for symptomatic relief. Over the course of the next month, her jaundice improved along with improvement in physical activity and appetite. Given the patient’s age, her functional capacity and poor prognosis related to the pancreatic adenocarcinoma, she was considered not to be a candidate for either chemotherapy or irradiation. The patient and her family received counselling from the palliative care team for initiation of hospice services.

## Case 2

**Figure 3.**
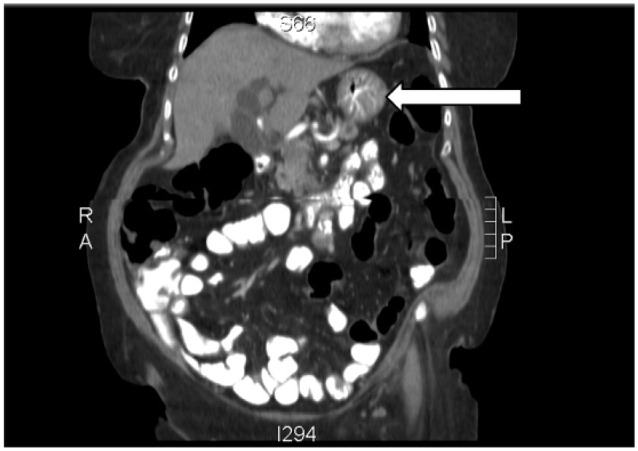
CT scan image of Case 1.

**Figure 4.**
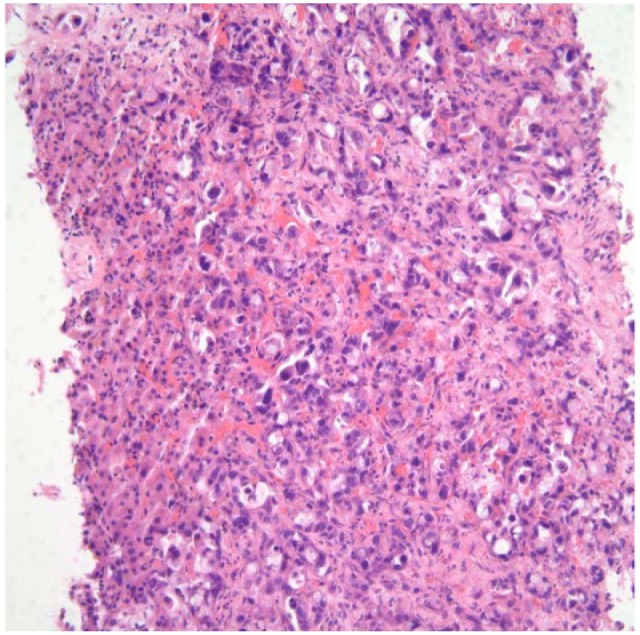
Pathology slide image of Case 2.

A 59-year-old man with a past medical history of left eye blindness due to glaucoma and a 1-year history of type 2 diabetes mellitus presented to the primary care clinic for a routine follow-up visit. He reported unintentional weight loss of 20 pounds in the prior 2 months with reduced appetite and fatigue. He also mentioned decreased interest in daily activities. The physical examination at this visit was unremarkable. Routine laboratory testing was performed, which revealed a serum glucose of 495 mg/dL and hemoglobin A1c (HgbA1c) of 13.0. A complete blood count, chemistry, and liver function tests were within normal limits (Table 2). A prior HgbA1c, measured 6 months earlier, was 7.5, and he had been placed on metformin 500 mg twice daily. Given his history of weight loss, a CT scan of the abdomen was performed to assess for an intraabdominal cause of weight loss. It revealed a lesion within the body of the pancreas with distal pancreatic duct dilatation, compatible with the presence of PDAC, along with multiple metastatic lesions within the liver (Figure 3). A CT-guided liver biopsy was performed, which revealed a poorly differentiated adenocarcinoma of pancreatic origin (Figure 4). The patient followed up in oncology clinic after confirmation of the diagnosis and was informed about the aggressive nature of the disease. He had a good functional status and was independent in all activities of daily living without any restrictions. The patient and his family were informed that chemotherapy would be palliative to extend survival while preserving his quality of life as much as possible. The patient opted for chemotherapy and was started on 28-day cycles of gemcitabine/paclitaxel.

**Table 2.** Laboratory Parameters of Case 2.

Parameter (Normal Range)	Six Months Earlier	At the Time of Diagnosis
Hemoglobin A1c (3.9% to 6.9%)	7.5	13
Glucose (70-105 mg/dL)	97	495
Aspartate transaminase (1-40 U/L)	28	31
Alanine transaminase (1-40 U/L)	42	34
Alkaline phosphatase (30-115 U/L)	76	115
Total bilirubin (0.1-1.2 mg/dl)	0.5	0.5

## Review of Literature

The association between diabetes mellitus and PDAC is well-established epidemiologically.^1,2^ While long-standing type 2 diabetes mellitus is a modest risk factor for the development of PDAC,^1,3,4^ new-onset diabetes has also been observed to be an early manifestation of PDAC.^5^

About 80% of patients with PDAC have either impaired glucose tolerance or type 2 diabetes at the time of diagnosis.^6^ A meta-analysis conducted by Ben et al in 2011 concluded that patients with diabetes had a 2-fold increased risk of developing PDAC compared to nondiabetics. However, the risk of PDAC was inversely proportional to the duration of diabetes and was highest among patients who were diagnosed with diabetes less than 1 year before the detection of PDAC (relative risk [RR] = 5.38).^7^ A prior meta-analysis conducted by Huxley et al in 2005 showed similar results. Patients who were diagnosed with diabetes less than 4 years earlier had a 50% higher risk of developing PDAC than patients who had diabetes for more than 5 years.^2^ The most recent meta-analysis by Batabyal in 2014 confirmed the aforementioned results, stating that although long-standing diabetes places patients at a risk of acquiring PDAC (RR = 1.36), the association is much higher for patients diagnosed with diabetes less than 1 year before diagnosis of PDAC (RR = 6.69).^8^ These studies suggest that new-onset diabetes mellitus or worsening glycemic control in a patient with previously controlled diabetes could be an indicator for PDAC. This is further supported by the fact that patients with newly diagnosed diabetes mellitus who underwent surgery for PDAC had improvement in their blood glucose levels post resection.^9,10^

While still incompletely understood, there are many theories proposed explaining the association between diabetes and PDAC. These can be broadly classified as metabolic, hormonal, and immunological alterations.^1^ Insulin resistance is the hallmark of diabetes, specifically type 2 diabetes mellitus. In response to this resistance, there is an oversecretion of insulin, which leads to an increase in β-cell mass. Experimental evidence suggests that insulin promotes cell proliferation through its mitogenic effects while simultaneously increasing glucose utilization by cells.^11^ Both these traits are inherent to tumor development. Thus, the exocrine pancreas in hyperinsulinemic patients is chronically exposed to high levels of this potential carcinogen. Furthermore, insulin, by its action on liver metabolism, upregulates levels of insulin-like growth factor-1 (IGF-1), which is known for its potent mitogenic and anti-apoptotic activities.^12-15^ While no epidemiological evidence has irrefutably linked PDAC risk and plasma levels of insulin-like growth factors, a case-control study indicated a possible association between polymorphic variants of the gene encoding IGF-1 and a risk of PDAC.^16^ More recent evidence suggests that inflammation plays an important role in the development of PDAC.^17^ Glucose and fat intake induce inflammation by increasing oxidative stress, which in turn increases insulin resistance.^18^ Multiple genome-wide studies have implicated that some of the genetic variations and loci that modify the risk for diabetes mellitus, have also been implicated in cell differentiation and development.^19^ A lower incidence of PDAC has been shown in patients with diabetes treated with metformin as opposed to those treated with insulin.^20-24^ This is believed to be a consequence of metformin reducing hepatic glucose output and hence circulating insulin levels.

While these theories attempt to explain how hyperglycemia predisposes to PDAC, the mechanism of development of diabetes in patients with PDAC is yet unknown. In vitro studies demonstrating intracellular defects in insulin action, and decreased glycogen synthase activity resulting in impaired glucose disposal have been the suggested mechanisms for PDAC-induced insulin resistance.^25,26^ A study by Basso et al suggested that a putative PDAC-associated diabetogenic factor could be a 2030-MW peptide that was observed in sera from pancreatic cancer patients and pancreatic cancer cell conditioned media.^27^ Adrenomedullin is thought to be another protein secreted by cancerous cells causing β-cell dysfunction and thereby leading to the development of diabetes in patients with PDAC.^28^ However, studies with larger cohorts need to be conducted to confirm its diagnostic value. Inasmuch as it is yet not possible to distinguish between type 2 diabetes mellitus from diabetes associated with PDAC in its early stages, multiple studies are currently underway in a quest to identify biomarkers that may be able to identify persons at high risk for developing PDAC.^29,30^

Our review suggests that it may be prudent to consider an underlying pancreatic malignancy when evaluating patients with an unexplained acute worsening of glycemic control and in patients with sudden-onset HHS in the appropriate clinical setting.

## References

[1] Li D. Diabetes and pancreatic cancer. Mol Carcinog. 2012;51:64-74. 22162232 10.1002/mc.20771 PMC3238796

[2] Huxley R Ansary-Moghaddam A Berrington de González A Barzi F Woodward M. Type-II diabetes and pancreatic cancer: a meta-analysis of 36 studies. Br J Cancer. 2005;92:2076-2083. 15886696 10.1038/sj.bjc.6602619 PMC2361795

[3] Fisher WE. Diabetes: risk factor for the development of pancreatic cancer or manifestation of the disease? World J Surg. 2001;25:503-508. 11396427 10.1007/s002680020344

[4] Noy A Bilezikian JP. Clinical review 63: Diabetes and pancreatic cancer: clues to the early diagnosis of pancreatic malignancy. J Clin Endocrinol Metab. 1994;79:1223-1231. 7962312 10.1210/jcem.79.5.7962312

[5] Wang F Herrington M Larsson J Permert J. The relationship between diabetes and pancreatic cancer. Mol Cancer. 2003;2:4. 12556242 10.1186/1476-4598-2-4 PMC149418

[6] Pannala R Leirness JB Bamlet WR Basu A Petersen GM Chari ST. Prevalence and clinical profile of pancreatic cancer-associated diabetes mellitus. Gastroenterology. 2008;34:981-987. 10.1053/j.gastro.2008.01.039 PMC2323514 18395079

[7] Ben Q Xu M Ning X Diabetes mellitus and risk of pancreatic cancer: a meta-analysis of cohort studies. Eur J Cancer. 2011;47:1928-1937. 21458985 10.1016/j.ejca.2011.03.003

[8] Batabyal P Vander Hoorn S Christophi C Nikfarjam M. Association of diabetes mellitus and pancreatic adenocarcinoma: a meta-analysis of 88 studies. Ann Surg Oncol. 2014;21:2453-2462. 24609291 10.1245/s10434-014-3625-6

[9] Permert J Ihse I Jorfeldt L von Schenck H Arnquist HJ Larsson J. Improved glucose metabolism after subtotal pancreatectomy for pancreatic cancer. Br J Surg. 1993;80:1047-1050. 8402064 10.1002/bjs.1800800841

[10] Fogar P Pasquali C Basso D Diabetes mellitus in pancreatic cancer follow-up. Anticancer Res. 1994;14:2827-2830. 7532931

[11] Ding XZ Fehsenfeld DM Murphy LO Permert J Adrian TE. Physiological concentrations of insulin augment pancreatic cancer cell proliferation and glucose utilization by activating MAP kinase, PI3 kinase and enhancing GLUT-1 expression. Pancreas. 2000;21:310-320. 11039477 10.1097/00006676-200010000-00014

[12] Bergmann U Funatomi H Yokoyama M Beger HG Korc M. Insulin-like growth factor I overexpression in human pancreatic cancer: evidence for autocrine and paracrine roles. Cancer Res. 1995;55:2007-2011. 7743492

[13] Ohmura E Okada M Onoda N Insulin-like growth factor I and transforming growth factor alpha as autocrine growth factors in human pancreatic cancer cell growth. Cancer Res. 1990;50:103-107. 2152769

[14] Stoeltzing O Liu W Reinmuth N Regulation of hypoxia-inducible factor-1alpha, vascular endothelial growth factor, and angiogenesis by an insulin-like growth factor-I receptor autocrine loop in human pancreatic cancer. Am J Pathol. 2003;163:1001-1011. 12937141 10.1016/s0002-9440(10)63460-8 PMC1868239

[15] Zeng H Datta K Neid M Li J Parangi S Mukhopadhyay D. Requirement of different signaling pathways mediated by insulin-like growth factor-I receptor for proliferation, invasion, and VPF/VEGF expression in a pancreatic carcinoma cell line. Biochem Biophys Res Commun. 2003;302:46-55. 12593846 10.1016/s0006-291x(03)00107-4

[16] Suzuki H Li Y Dong X Hassan MM Abbruzzese JL Li D. Effect of insulin-like growth factor gene polymorphisms alone or in interaction with diabetes on the risk of pancreatic cancer. Cancer Epidemiol Biomarkers Prev. 2008;17:3467-3473. 19064563 10.1158/1055-9965.EPI-08-0514 PMC2600618

[17] Greer JB Whitcomb DC. Inflammation and pancreatic cancer: an evidence-based review. Curr Opin Pharmacol. 2009;9:411-418. 19589727 10.1016/j.coph.2009.06.011

[18] Busserolles J Rock E Gueux E Mazur A Grolier P Rayssiguier Y. Short-term consumption of a high-sucrose diet has a pro-oxidant effect in rats. Br J Nutr. 2002;87:337-342. 12064343 10.1079/BJNBJN2002524

[19] Duffy DL. Genetic determinants of diabetes are similarly associated with other immune-mediated diseases. Curr Opin Allergy Clin Immunol. 2007;7:468-474. 17989522 10.1097/ACI.0b013e3282f1dc99

[20] Bowker SL Majumdar SR Veugelers P Johnson JA. Increased cancer-related mortality for patients with type 2 diabetes who use sulfonylureas or insulin. Diabetes Care. 2006;29:254-258. 16443869 10.2337/diacare.29.02.06.dc05-1558

[21] Monami M Lamanna C Balzi D Marchionni N Mannucci E. Sulphonylureas and cancer: a case-control study. Acta Diabetol. 2009;46:279-284. 19082520 10.1007/s00592-008-0083-2

[22] Libby G Donnelly LA Donnan PT Alessi DR Morris AD Evans JM. New users of metformin are at low risk of incident cancer: a cohort study among people with type 2 diabetes. Diabetes Care. 2009;32:1620-1625. 19564453 10.2337/dc08-2175 PMC2732153

[23] Currie CJ Poole CD Gale EA. The influence of glucose-lowering therapies on cancer risk in type 2 diabetes. Diabetologia. 2009;52:1766-1777. 19572116 10.1007/s00125-009-1440-6

[24] Landman GW Kleefstra N van Hateren KJ Groenier KH Gans RO Bilo HJ. Metformin associated with lower cancer mortality in type 2 diabetes: ZODIAC-16. Diabetes Care. 2010;33:322-326. 19918015 10.2337/dc09-1380 PMC2809274

[25] Liu J Knezetic JA Strömmer L Permert J Larsson J Adrian TE. The intracellular mechanism of insulin resistance in pancreatic cancer patients. J Clin Endocrinol Metab. 2000;85:1232-1238. 10720068 10.1210/jcem.85.3.6400

[26] Isaksson B Strömmer L Friess H Impaired insulin action on phosphatidylinositol 3-kinase activity and glucose transport in skeletal muscle of pancreatic cancer patients. Pancreas. 2003;26:173-177. 12604916 10.1097/00006676-200303000-00014

[27] Basso D Valerio A Seraglia R Putative pancreatic cancer-associated diabetogenic factor: 2030 MW peptide. Pancreas. 2002;24:8-14. 11741177 10.1097/00006676-200201000-00002

[28] Aggarwal G Ramachandran V Javeed N Adrenomedullin is up-regulated in patients with pancreatic cancer and causes insulin resistance in beta cells and mice. Gastroenterology. 2012;143:1510-1517.e1. 22960655 10.1053/j.gastro.2012.08.044 PMC3787599

[29] Satake K Takeuchi T Homma T Ozaki H. CA19-9 as a screening and diagnostic tool in symptomatic patients: the Japanese experience. Pancreas. 1994;9:703-706. 7846012 10.1097/00006676-199411000-00005

[30] Kim JE Lee KT Lee JK Paik SW Rhee JC Choi KW. Clinical usefulness of carbohydrate antigen 19-9 as a screening test for pancreatic cancer in an asymptomatic population. J Gastroenterol Hepatol. 2004;19:182-186. 14731128 10.1111/j.1440-1746.2004.03219.x

